# The Yin and Yen of GABA in Brain Development and Operation in Health and Disease

**DOI:** 10.3389/fncel.2012.00045

**Published:** 2012-11-09

**Authors:** Yehezkel Ben-Ari

**Affiliations:** ^1^Institut National de la Santé et de la Recherche MédicaleMarseille, France

GABA (A) receptor mediated signals have many facets and a plethora of expression mechanisms and consequences on the operation of behaviorally relevant patterns. Starting from the simple view of inhibition/hyperpolarization, the actions of GABA revealed to be far more complex with the identification of interneuronal types, their projections, how they modulate brain patterns, and the crucial roles they serve in health and disease.

As in other fields of neuroscience, recent emphasis has been devoted to gaining a better understanding of the development of GABA signals: developmental neurobiology has invaded all fields of brain research. In this e-book, along the line of a wide range of investigations stressing the roles of GABA in cell proliferation, Young et al. (2010) report their results on GABA actions in the sub ventricular zone. GABAergic interneurons follow a long journey to their assigned targets and we are beginning to know how they are controlled during that journey. Friocourt and Parnavelas (2010) discuss the role of ARX and Livnat et al. (2010) pursue their importance contributions in this domain analyzing the PAF-AH catalytic subunits modulate the Wnt pathway.

Extensive investigations have been devoted to the trophic roles exerted by GABA during maturation. Sernagor et al. (2010) analyze the role of GABAergic signals in development and adult neurogenesis stressing the similarities and differences between different neuronal systems. An interesting twist to these studies is provided by Heistek et al. (2010) showing that maturation of Gaba currents and associated voltage gated currents differ in different strains of rodents leading also to different oscillations generated by networks. This important contribution illustrates how the intrinsic features of GABAergic synaptic currents impact brain operation. This is also analyzed in a different perspective by Baltz et al. (2010) showing that Gaba interneurons play a crucial role in the maturation of brain patterns *in vitro* including the well investigated Giant Depolarizing Potentials (GDPs) that have been identified in developing networks in a wide range of brain preparations and structures during early developmental stages. Somatic projecting interneurons have different brain patterns that are crucial in the generation of behaviorally relevant oscillations. Ellender and Paulsen (2010) analyze these tunes, their generation mechanisms and consequences on the network. Cortical neurons *in vivo* may operate in high-conductance states, due to synaptic activity that are sometimes several-fold larger than the resting conductance. Destexhe (2010) analyze how the contribution of inhibition in such high-conductance states.

The Depolarizing/hyperpolarizing shift during maturation is a fundamental features of brain maturation that along other developmental sequences illustrates how developing currents and signaling in general have a different agenda than adult ones. Excitatory GABA plays a crucial role in the trophic roles that GABA exerts on all developmental mechanisms from proliferation to differentiation, growth, and synapse formation. These features are analyzed here by several groups. Ormond and Woodin (2011) pursue their analysis of the phenomenon of paired pre- and postsynaptic activity in area CA1 of the hippocampus that induces long-term inhibitory synaptic plasticity at GABAergic synapses. These long-term changes in the driving force of GABA is a vivid illustration of how activity impacts brain networks via alterations of intracellular levels of chloride. It is usually assumed that the low levels of Cl in immature neurons is due to the lack of expression of the chloride exporter KCC2, here Khalilov et al. (2011) show that in KCC2 Kos that die at birth, networks generate seizures suggesting that this device is needed in embryos. Valeeva et al. (2010) show how this depolarization that does not reach spike threshold yet can generate action potentials by activating a voltage gated current illustrating the important convergence of synaptic and voltage gated currents. An important conceptual issue receives in the work of Dehorter et al. (2011) an elegant reply. Indeed, if immature patterns generate different patterns they must at some stage shift to adult patterns to enable a smooth passage from patterns devoted to growth and patterns that control behaviors and motricity. Here, the authors show that Medium Spiny neurons that must be quite silent to enable the generation of targeted movement are silenced precisely when the pup start generating these movements; this is mediated by alterations of NMDA receptor mediated currents and voltage gated currents stressing the importance and precision of the timing. Sale et al. (2010) provide two important contributions on the control of plasticity by GABA in the maturation of the visual system and the E/I balance in amblyopia stressing the possible therapeutic perspectives of early corrections of inhibition in visual acuity.

Finally, Nardou et al. (2011) show how Phenobarbital but not diazepam exert a direct action on AMPA/kainate mediated currents illustrating how classical GABA acting drugs used for generations as classical tools to dissect GABA signals can have diverse actions. Cesetti et al. (2012) analyze the roles of GABA in the developmental changes of GABAergic mechanisms in human visual cortex across the lifespan.

Summing up, these studies illustrate vividly the importance of GABAergic signals and how they genuinely control brain operation from the earliest developmental stages sculpting neuronal shapes, controlling their intrinsic features, and determining how they operate in health and disease.
